# Reducing major lower extremity amputations after the introduction of a multidisciplinary team in patient with diabetes foot ulcer

**DOI:** 10.1186/s12902-016-0111-0

**Published:** 2016-07-07

**Authors:** Chuan Wang, Lifang Mai, Chuan Yang, Dan Liu, Kan Sun, Weidong Song, Baoming Luo, Yan Li, Mingtong Xu, Shaoling Zhang, Fangping Li, Meng Ren, Li Yan

**Affiliations:** Department of Endocrinology and Metabolism, Sun Yat-sen Memorial Hospital, Sun Yat-sen University, Guangzhou, 510120 China; Department of Orthopaedic Surgery, Sun Yat-sen Memorial Hospital, Sun Yat-sen University, Guangzhou, 510120 China; Department of Ultrasonography, Sun Yat-sen Memorial Hospital, Sun Yat-sen University, Guangzhou, 510120 China

**Keywords:** Diabetes foot, Ulcer, Amputation

## Abstract

**Background:**

Diabetic foot ulceration is receiving more attention because of its high amputation and mortality rate. It is essential to establish the frequency of amputations in people with diabetes after any change to the management of diabetic foot care. The present study aim to compare the frequency of lower-extremity amputations in patients with diabetes foot ulcer over a ten-year period.

**Methods:**

Six hundred forty eight patients with diabetes foot ulcer were retrospectively studied from 2004 to 2013. The clinical features, laboratory results and the lower-extremity amputations were recorded. Major amputation was defined as amputations above the ankle while minor amputation was amputations below the ankle in the present study.

**Results:**

Patients with diabetic foot ulcer were old (age 66.96 ± 11.96 years), with a long duration of diabetes (10.30 ± 6.94 years), high HbA1c (9.19 ± 2.62 %), SBP (144.05 ± 24.18 mmHg), DBP (79.53 ± 11.88 mmHg), LDL-C (2.71 ± 0.93 mmol/L) and had great frequency of neuropathy (62.7 %), retinopathy (45.0 %), nephropathy (39.5 %) and PAD (33.2 %). From 2004 to 2013, the frequency of all lower-extremity amputations is 12.0 % (5.2 % major amputation, 6.8 % minor amputation). The frequency of major amputations decreased from 9.5 % in 2004 and 14.5 % in 2005 to less than 5.0 % after 2006. In particular, there was a significant decline in major amputations of diabetic foot patient with Wagner 3 to 4 wounds. The frequency rate of major amputations in diabetic foot patient with Wagner 3 to 4 wounds fell from 35.7 % in 2004 to 4.4 % after 2007. The change in frequency of minor amputations was fluctuation.

**Conclusion:**

This study demonstrates that the introduction of a multidisciplinary team, coordinated by an endocrinologist and a podiatrist, for managing diabetic foot disease is associated with a reduction in the frequency of major amputations in patients with diabetes.

## Background

In 2011, Chinese Diabetes Society studies have shown that Chinese diabetic foot disease accounted for 12.4 % of the hospitalized patients with diabetes in 2010 with a high amputation rate of 7.3 %, where amputation rates caused by diabetes accounted for 28.2 % of all amputations and 41.5 % of non-traumatic amputations, which stands first on the non-traumatic amputation list [1]. Foot ulceration is one of the most serious and disabling complications of diabetes mellitus. Diabetic foot ulceration (DFU) develops in 15 - 25 % of DM patients. It is the most common cause of nontraumatic foot amputation worldwide. Approximately 20 % of those cases require amputation [2, 3]. DFUs are complex, chronic wounds, which have a major long-term impact on the morbidity, mortality and quality of patients’ lives. Furthermore, DFU is responsible for substantial emotional and physical distress as well as productivity and financial losses that lower the quality of life [4]. Because of its high amputation and mortality rate, DFU is receiving more and more attention as one of the severe diabetes related complications.

As diabetes is a multi-organ systemic disease, all comorbidities that affect wound healing must be managed by a multidisciplinary team for optimal outcomes with DFU. Until now, numerous studies have shown that a multidisciplinary team can reduce amputation rates, lower costs, and leads to better quality of life for patients with DFU. A multidisciplinary team can decrease the risks associated with DFU and amputation by 50 - 85 % [5, 6]. Since the early 2004, a multidisciplinary team including nurse, orthopedics, plastic surgery, vascular surgery, and nutritional department, headed by endocrinology department has been developed in our Hospital. Education, blood sugar control, wound debridement, advanced dressing, offloading, surgery, and advanced therapies were used clinically and comprehensively from 2004 and reinforced from 2006. So establishing the frequency of amputations in people with diabetes after any change to the management of diabetic foot care is essential, and it is necessary to know how rates change over time.

The aim of this study was to analyze the effect of the introduction of multidisciplinary diabetic foot team in our hospital, by evaluating trends in major and minor lower-extremity amputation rate in people with DFU in our department between 2004 and 2013.

## Methods

### Study population

Medical records of the diabetic inpatients admitted to the Diabetic Foot Care Center, Department of Endocrinology and Metabolism at the *Sun Yat-sen Memorial Hospital, Sun Yat-sen University* from 1 April 2004 to 31 December 2013 were retrospectively reviewed. Foot ulcer was graded according to Wagner’s classification [7]: Grade 0, high-risk foot; Grade 1, superficial ulcer; Grade 2, deep ulcer penetrating to tendon, bone, or joint; Grade 3, deep ulcer with abscessor osteomyelitis; Grade 4, localized gangrene; and Grade 5, extensive gangrene requiring a major amputation. Totally 648 patients with diabetic foot ulcer at Wagner Grade 1-5 were recruited with 334 males and 314 females. Among all the 648 patients, 78 patients underwent amputation.

### Clinical and biochemical measurements

Detailed records of these patients were retrieved and available information, including clinical history, laboratory profiles, therapeutic course and outcome, were collected. Age, duration of diabetes and diabetes complications were recorded. Blood pressure was measured. Laboratory test results, including fasting blood glucose, glycosylated hemoglobin (HbA1c) and blood lipids were tested.

Diabetic retinopathy is detected during an eye examination by ophthalmologist that includes stages 1-4 pathological change (0 = no retinopathy, 1 = mild non-proliferative retinopathy, 2 = moderate non-proliferative retinopathy, 3 = severe non-proliferative retinopathy, and 4 = proliferative retinopathy). Diabetic patients with urinary albumin to creatinine ratio greater than 30 mg/g or 24-hour urinary albumin greater than 0.5 g or with elevated serum creatinine were defined as having diabetic nephropathy. Diabetic peripheral neuropathy is the diagnosis by comprehensive evaluation of the appearance of the feet, presence of ulceration, ankle reflexes, large fiber neuropathy, pressure sensation and nerve conduction tests. The patient is diagnosed with peripheral arterial disease (PAD) when the ankle brachial index is ≤ 0.90 or absence of dorsalis pedis artery pulse or ultrasonography in detecting lower extremity arterial stenosis greater than 50 %. Amputation was defined as the complete loss of the transverse anatomical plan of any part of the lower limb. All amputations performed below the ankle were defined as minor amputations, whereas amputations above the ankle were defined as major.

In patients with more than one procedure, the highest level of amputation was analyzed. In cases where patients underwent multiple amputations during the study period, the amputation that was deemed to have the largest impact on the patient’s quality of life was used for the analysis. When patients underwent several amputations on the same level during an extended period of time, the first recorded amputation was used. Repeated amputations (re-amputations) performed within the same period of hospital care, due to poor healing or infection, were classified as the last reported amputation within this period. Re-amputations and subsequent amputations of a contralateral limb (double amputations) were analyzed separately.

### Statistical analysis

All calculations were performed using SPSS version 13.5 software (SPSS Inc., Chicago, IL, USA). The data were expressed as means ± SD when variables were normally distributed. Frequencies and percentages were used for statistical descriptions. Continuous variables were analyzed using one way ANOVA. Frequency data were examined by χ^2^ test. Statistical significance was defined as *P* < 0.05.

## Results

### Clinical characteristics of study subjects

The average age of patients with DFU was 66.96 ± 11.96 years, with mean diabetes duration of 10.30 ± 6.94 years. The overall glucose level was high, with an average HbA1c of 9.19 ± 2.62 %. Among all the diabetic foot patients, the frequency of neuropathy, retinopathy, nephropathy and PAD were 62.67 %, 45.00 %, 39.47 % and 33.24 %, respectively.

### Clinical pathway of standardized and multidisciplinary team management

**Fig. 1:**
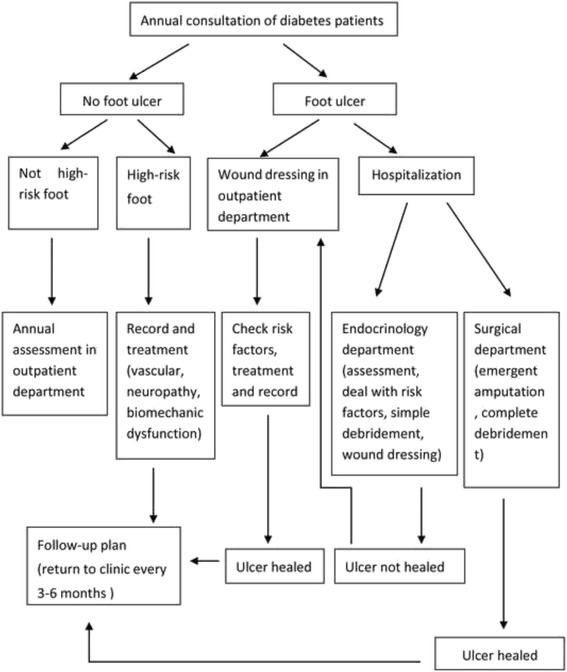
Standardized management pathway of diabetic foot patients

**Fig. 2:**
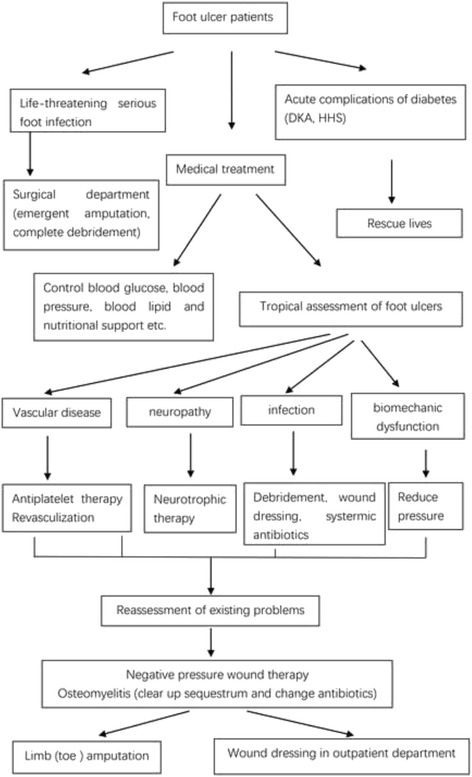
Clinical pathway of diabetic foot ulcer (and or gangrene) patients in department of endocrinology

Standardized management pathway of diabetic foot patients before the multidisciplinary team was shown in Fig. 1. A multidisciplinary team headed by endocrinology department was reinforced in our hospital after year 2006, which including nurse, orthopedics, plastic surgery, vascular surgery, and nutritional department. Clinical pathway of patients with diabetic foot ulcer in department of endocrinology after introduction of a multidisciplinary team was shown in Fig. 2.

### Demographic and clinical data of DFU patients

We then compared the demographic and clinical data of DFU patients in each year. No significant difference was found in age, diabetes duration, SBP, DBP, HbA1c, TC, LDL-C, TG, HDL-C or creatinine of the patients in each year (*p* > 0.05) during 2004-2013. Whereas, there was significant difference in hospital days (*p* = 0.015), which showed an overall downward trend (Table 1).

**Table 1:** The clinical characteristics of patients with DFU from 2004 to 2013

	2004	2005	2006	2007	2008	2009	2010	2011	2012	2013	*p*
number	42	55	36	56	73	54	110	72	54	96	
Age (years)	67.25±11.42	68.10±11.68	70.24±9.27	68.76±10.88	67.40±11.60	68.43±12.25	66.52±12.32	64.35±13.13	65.62±11.85	66.14±12.41	0.324
Duration of diabetes (years)	8.83±5.00	9.40±5.98	10.42±6.80	9.75±6.78	11.20±8.1	8.82±5.47	11.34±7.54	10.11±7.76	9.64±6.07	10.1±6.78	0.297
SBP (mmHg)	142.92±25.07	141.04±22.22	145.76±27.39	142.61±21.76	138.88±22.92	145.59±21.89	141.13±19.27	140.76±23.52	142.70±24.60	140.30±20.50	0. 36
DBP(mmHg)	80.32±11.73	79.96±14.60	78.32±10.41	80.68±10.78	79.21±9.48	79.04±14.23	78.07±13.79	79.19±9.79	80.34±12.73	79.84±9.97	0.87
BMI (kg/m^2^)	20.80±6.16	19.63±8.55	20.83±7.87	21.62±5.45	20.09±7.38	21.35±7.13	22.15±6.43	20.49±6.32	20.74±7.57	21.90±5.93	0.744
HbAlc (%)	9.60±3.09	8.88±2.50	9.16±2.33	10.55±4.40	8.86±2.12	9.54.±2.72	9.01±2.67	8.97±2.45	9.20±3.42	8.87±3.02	0.92
LDL-C (mmol/L)	2.47±0.91	2.83±1.10	2.65±0.75	2.77±0.78	2.94±0.98	2.51±0.92	2.43±0.87	2.66±0.91	2.69±0.91	2.80±1.13	0.06
TG (mmol/L)	1.29±1.04	1.47±1.31	1.39±0.87	1.69±1.74	1.57±0.99	1.63±1.66	1.39±0.74	1.54±1.34	1.75±1.43	1.64±1.27	0.30
HDL-C (mmol/L)	1.08±0.37	1.02±0.36	0.97±0.32	1.10±0.38	1.08±0.93	097±0.25	0.98±0.35	1.12±0.40	1.04±0.78	0.98±0.33	0.45
Cr (μmol/L)	136.08±111.97	129.92±96.19	128.61±111.14	129.02±81.71	125.98±64.70	131.08±68.28	123.53±85.15	130.02±70.08	129.83±89.75	127.67±90.27	0.98
Hospital days (days)	32.62±26.24	31.00±28.34	26.57±29.05	24.30±18.20	26.57±21.69	21.20±17.79	20.75±13.10	19.37±11.17	21.46±14.70	20.16±15. 35	0.015

*SBP* systolic blood pressure, *DBP* diastolic blood pressure, *BMI* body mass index, *TG* triglycerides, *TC* total cholesterol, *HDL-C* high-density lipoprotein cholesterol, *LDL-C* low-density lipoprotein cholesterol, *Cr* serum creatinine

The demographic and biochemical characteristics of the study subjects before and after the introduction of a multidisciplinary team are shown in Table 2. Average hospital days of patients are the key indication which reflects the working efficiency of the management team. As shown in Table 2, although most measures were not different before and after multidisciplinary team introduction, the average hospitalization day is significantly reduced after the multidisciplinary collaboration DF management team was reinforced in the year 2006.

**Table 2:** Characteristics of study patients Wagner grade 3-4 before and after introduction of multidisciplinary team

	Before multidisciplinary team	After multidisciplinary team	*p*
Duration of diabetes (years)	8.55±5.49	10.30±7.05	0.081
SBP (mmHg)	142.62±23.58	142.65±23.81	0.995
DBP (mmHg)	76.16±12.30	79.34±12.07	0.128
BMI	19.52±7.73	21.17±6.17	0.183
Cr (μmol/L)	118.87±80.39	120.63±58.94	0.886
HbAlc (%)	9.84±2.52	9.67±2.69	0.771
TC (mmol/L)	4.22±0.98	4.22±1.15	0.992
LDL-C (mmol/L)	2.70±1.02	2.68±0.89	0.931
TG (mmol/L)	1.14±0.74	1.53±1.50	0.074
HDL-C (mmol/L)	0.97±0.35	1.09±1.11	0.483
Hospital days (days)	41.29±32.76	24.01±19.21	0.000

*SBP* systolic blood pressure, *DBP* diastolic blood pressure, *BMI* body mass index, *Cr* serum creatinine, *TG* triglycerides, *TC* total cholesterol, *HDL-C* high-density lipoprotein cholesterol, *LDL-C* low-density lipoprotein cholesterol

### Wagner grade distribution of DF patients

From 2004 to 2013, proportions of patients at Wagner grade 1-4 are 9.72 %, 37.81 %, 26.85 %, 23.92 % and 1.70 % respectively. We analyzed Wagner grade distribution in each year and found them significantly different from one another (*P* < 0.01). Furthermore, we also tested distribution of patients at Wagner 3-5 as well as Wagner 3-4, and found no significant difference between each year (*p* > 0.05). But when it came to patients at Wagner 1-2, the distribution had significant difference (*P* < 0.01).

### Amputation rate analysis of DFU patients

**Fig. 3:**
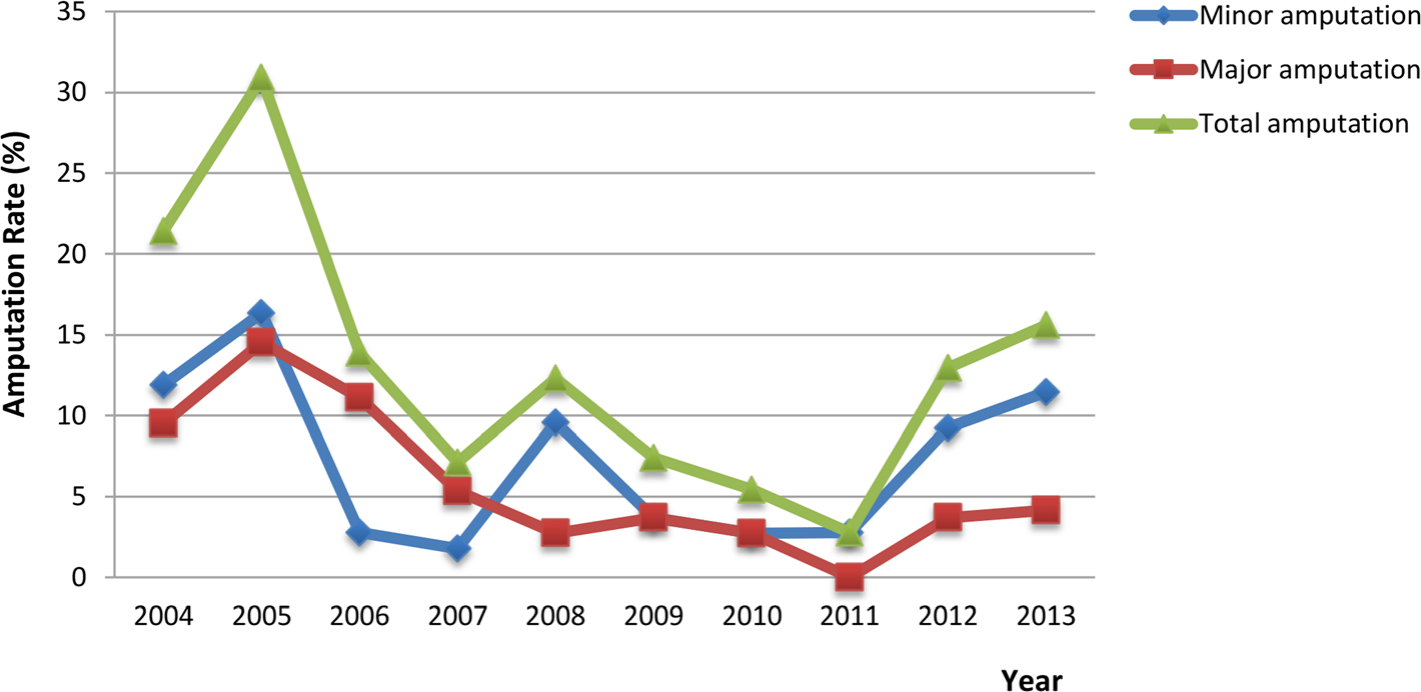
The amputation rate analysis of DFU patients from 2004 to 2013 (Major amputation: amputations above the ankle; Minor amputation: amputations below the ankle)

The average annual amputation rate from 2004 to 2013 was 12.03 %, among which major amputation took 5.24 % and minor amputation took 6.79 %. When being analyzed by year, major amputation changed from 9.52 %-14.54 % before 2006 to below 5 % after 2006. Meanwhile, minor amputation fell from above 10 % (from 11.90 % to 16.36 %) before 2006 to below 10 % (from 2.72 % to 9.58 %) after 2006, but raised back to 11.45 % in 2013 (Fig. 3).

### Amputation rates in different Wagner Grade

Among the 308 patients at Wagner grade 1-2 from 2004 to 2013, 6 underwent amputation, including 2 minor amputations and 1 major amputation in 2004, 2 minor amputations in 2012, and 1 minor amputation in 2013. There was no amputation in the rest of the years. Patients at Wagner grade 5 during the 10 years added up to 11, among whom 4 underwent minor amputations and 2 suffered from major amputations. The amputation rate in hospital was 54.55 %.

**Fig. 4:**
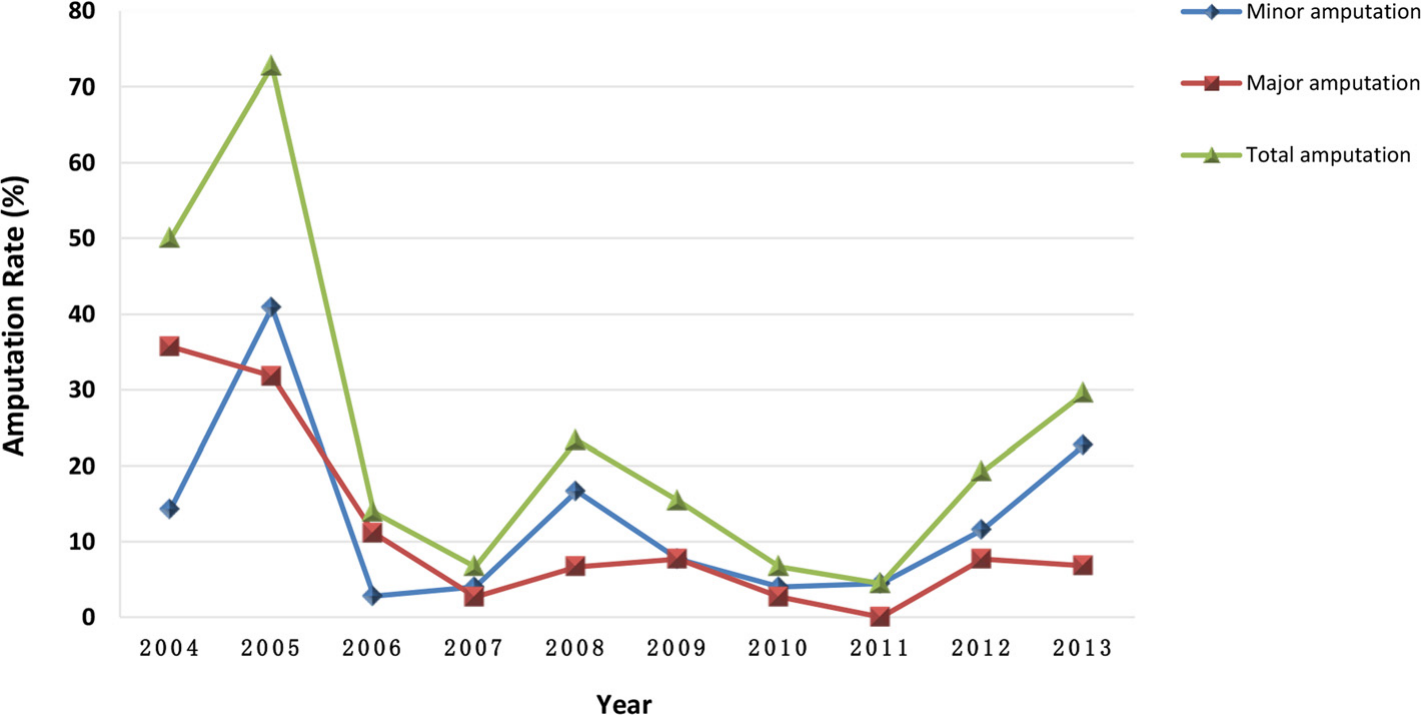
Amputation rates in patients at Wagner grade 3-4 across time (Major amputation: amputations above the ankle; Minor amputation: amputations below the ankle)

During 2004-2013, major amputation rate of DFU patients at Wagner grade 3-4 significantly reduced in 2006 and stayed below 5 % since then (Fig. 4). Minor amputation rate also went down in 2006, but somehow appeared a little rebound later. Meanwhile, statistical analysis of clinical data of all the patients showed a downtrend in length of hospital days (*P* = 0.001), but no significant difference was found in age, diabetes duration, SBP, DBP, HbA1c, TC, LDL-C, TG, HDL-C and creatinine levels.

## Discussion

In the present study, we found that the introduction of a multidisciplinary team in the treatment of DFU was associated with a reduction in the frequency of major amputations in patients with diabetes. This study is the first from China to report a reduction in major amputations following introduction of a multidisciplinary diabetic foot team.

The amputation risk varied according to different condition of the diabetic foot. Patients at Wagner grade 1-2 had relatively mild foot problem so as the amputation rate was low and prognosis was good. However, patients at Wagner grade 3-5 suffered from more severe condition as well as high amputation risk and great harm [2, 3]. According to their Wagner grades, we analyzed the amputation rate of DFU patients. During 2004-2013, the amputation rate of DFU patients at Wagner grade 1-2 was 1.95 %, with no changing in the last 10 years. At the same time, amputation rate of patients at Wagner grade 3-4 was 19.47 % and changed greatly during the last 10 years.

Another interesting phenomenon we found in the study was that minor amputation (below the ankle) of DF patients at Wagner grade 3-4 also showed a downward trend, but appeared significant fluctuation. Krishnan [8] and Jorgensen [9] et al. observed similar phenomenon in diabetes patients that major amputation rate decreased significantly while minor amputation had little change and obvious fluctuation. It might be attributed to the following reason that patients who used to need major amputation operation acquired lower amputation level after receiving proper treatment, making minor amputation take place of the major amputation, which definitely proved the effectiveness of our management

Based on the previous researches, there are many factors that determine the amputation rate [10–14]. Old age, smoking, diabetes duration, diabetes chronic complications as DN and DR, and other co-morbidities like hypertension are all risk factors of amputation of DF patients. The complicated condition of DF patients made the treatment really difficult, therefore brought long treatment period, high risk and high amputation rate. Therefore, our medical treatment was carried out based on those risk factors and in line with the recommendations of the International Consensus on the Diabetic Foot [15], and the trend of diabetic foot ulceration amputation appeared in our department was in accord with other counties reported in the corresponding period of time. Thus, we can draw the conclusion that disciplinary comprehensive management of DF patients is effective with no doubt.

To our great amazement, amputation rate of DFU patients in our department during 2004-2013 changed accordantly with our management level, especially in those at Wagner grade 3-4. In retrospect, the improvement of DF management in our department can be divided into two periods roughly.

From 2004, we began to set up standard management of DF patients in our department and explored a proper management pattern. In this period, we trained a medical team of passionate doctors and nurses who were skilled with DF patients’ treatment. Furthermore, we built DF workshop and accomplished plantar pressure measurement of normal people and diabetes mellitus patients. By means of new-type dressing, epidermal growth factors and therapeutic pressure-lowing footwear, we gradually built up multidisciplinary collaboration operation pattern. During this period, DFU patients’ major amputation rate reduced from 35.71 % in 2004, 31.82 % in 2005, to 11.11 % in 2006, especially in those at Wagner grade 3-4.

After 2006, the multidisciplinary collaboration DF management team was reinforced. Multidisciplinary consultation and collaboration operation pattern were built and treatment techniques like negative pressure wound therapy and ultrasonic debridement were introduced. We also pioneered the operation of lower extremity arterial interventional therapy in endocrinology department. Furthermore, clinical management pathway of DF disease was set up and improved with time. In this period of time, major amputation of DF patients at Wagner grade 3-4 appeared further reduction than years before 2006, as the average major amputation rate was 4.44 %.

This study has several limitations. First, the results of the present study only included Chinese subjects and might not be representative of other groups. Second, all invited participants in the present study were at the single-center level, which was a convenience sample and selection bias is inevitable. Third, although we did find that the introduction of a multidisciplinary team for managing diabetic foot disease is associated with a reduction in the frequency of major amputations, other potential mediators such as compliance of treatment, and prognostic evaluation such as hospitalization expenses and DFU mortality were not evaluated in the present study. Fourth, due to the retrospective design of the current study, we should cautiously interpret the present finding, especially because it is subject to substantial recall bias. Fifth, the present study only includes 648 diabetes patients, further prospective studies with larger sample size are needed to determine the precise value of multidisciplinary team in management of diabetic foot disease.

## Conclusion

In conclusion, this study demonstrates that the introduction of a multidisciplinary team for managing the diabetic foot, headed by endocrinology department is associated with a reduction in the frequency of major amputations in patients with diabetes in China.

## Abbreviations

DFU, diabetic foot ulceration; HbA1c, glycosylated hemoglobin; PAD, peripheral arterial disease; SBP, systolic blood pressure; DBP, diastolic blood pressure; TG, triglycerides; TC, total cholesterol; HDL-C, high-density lipoprotein cholesterol; LDL-C, low-density lipoprotein cholesterol
